# Supplementary material for: The interactome of intact mitochondria by cross-linking mass spectrometry provides evidence for coexisting respiratory supercomplexes
Source: Mol Cell Proteomics. 2017 Dec 8;17(2):216–32. doi: 10.1074/mcp.RA117.000470 (PMC5795388; doi:10.1074/mcp.RA117.000470)
Supplement: Supplemental Data [file supp_RA117.000470_133922_0_supp_23978_fzffwf.zip › spectra_annotation/mito_spectra_annotation/37-1-13-1-9-1.pdf]

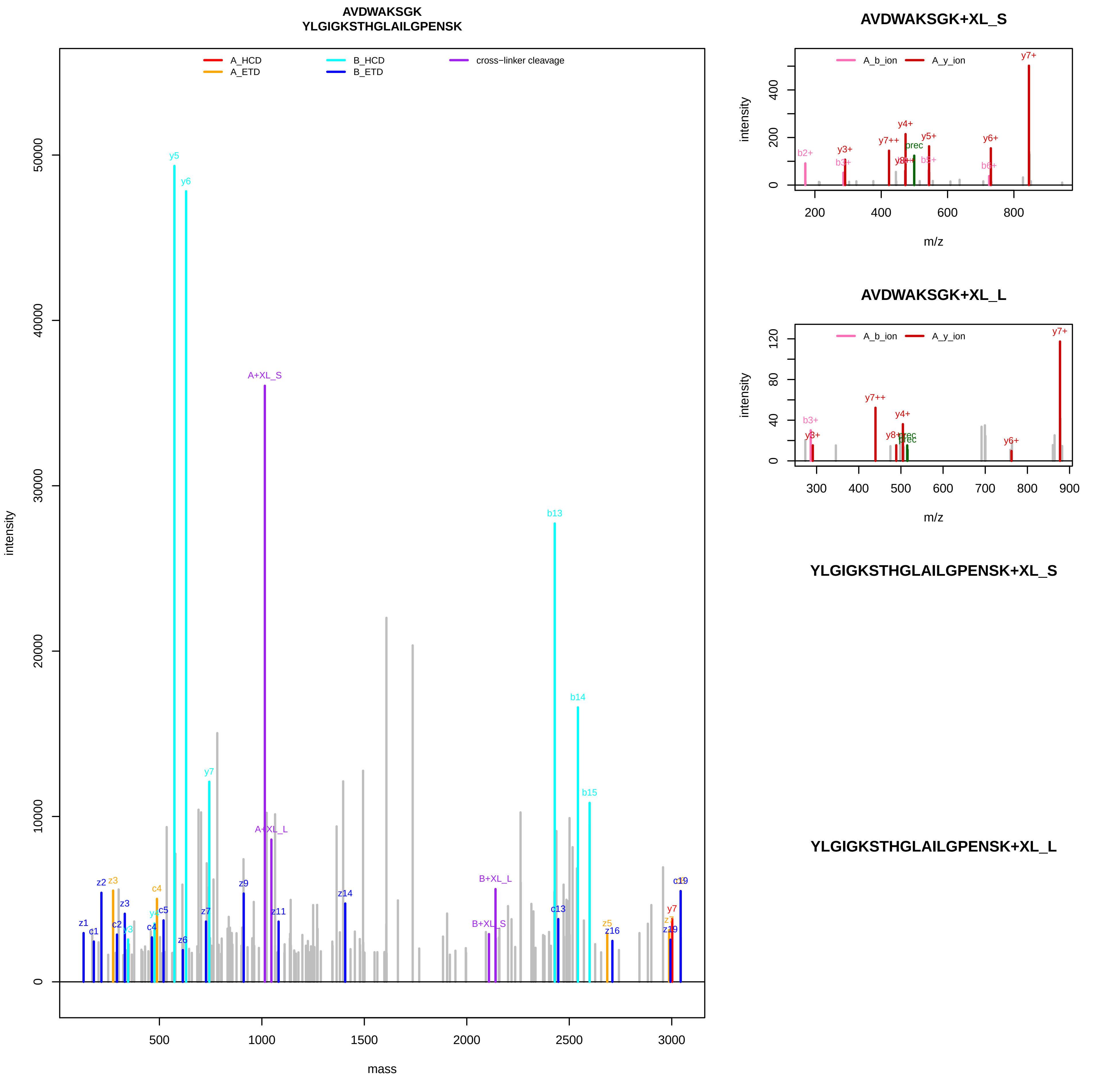
AVDWAKSGK
YLGIGKSTHGLAILGPENSK
A_HCD
A_ETD
B_HCD
B_ETD
cross−linker cleavage
intensity
mass
y5
y6
A+XL_S
A+XL_L
b13
b14
b15
y7
B+XL_L
B+XL_S
z1
c1
z2
z3
c2
c4
c5
z6
z7
z9
z11
z14
c13
z5
z16
z19
c19
AVDWAKSGK+XL_S
A_b_ion
A_y_ion
m/z
AVDWAKSGK+XL_L
YLGIGKSTHGLAILGPENSK+XL_S
YLGIGKSTHGLAILGPENSK+XL_L
